# Sodium-glucose cotransporter-2 inhibitor therapy improves renal and hepatic function in patients with cirrhosis secondary to metabolic dysfunction associated steatotic liver disease and type 2 diabetes

**DOI:** 10.3389/fendo.2025.1531295

**Published:** 2025-05-15

**Authors:** Alessandro Colletta, Katherine M. Cooper, Giuseppe Placentino, Deepika Devuni, Cosimo Colletta

**Affiliations:** ^1^ Division of Internal Medicine, UMass Chan Medical School, Division of Internal Medicine, Worcester, MA, United States; ^2^ Division of Gastroenterology, UMass Chan Medical School, Division of Internal Medicine, Worcester, MA, United States; ^3^ Diabetes Clinic, Azienda Sanitaria Locale Verbano Cusio Ossola (ASL VCO), Verbania, Italy; ^4^ Hepatology COQ, Madonna del Popolo Hospital, Omegna, Italy

**Keywords:** Child-Turcotte-Pugh B cirrhosis, sodium-glucose cotransporter-2 inhibitors, metabolic dysfunction associated steatotic liver disease, type 2 diabetes mellitus, glomerular filtration rate, chronic kidney disease

## Abstract

**Purpose:**

Metabolic dysfunction-associated steatotic liver disease (MASLD) increases the risk of chronic kidney disease (CKD), compounding morbidity in patients with cirrhosis. Sodium-glucose cotransporter-2 inhibitors (SGLT2i) are disease-modifying agents in type 2 diabetes mellitus (T2DM) and CKD, but studies on their use in cirrhosis are limited. We aimed to assess the effect of SGLT2i therapy on renal and hepatic function in patients with Child-Turcotte-Pugh (CTP) B cirrhosis and T2DM.

**Methods:**

We conducted a 48-month longitudinal, retrospective cohort study of 54 patients with CTP B cirrhosis secondary to MASLD and T2DM who were initiated on SGLT2i (n=27) or insulin (n=27). Laboratory data were collected every 3 months. Liver stiffness (LS) was measured every 6 months via transient elastography (TE) and acoustic radiation force impulse with shear wave velocity (ARFI-SWV). The primary outcome was change in glomerular filtration rate (GFR) and chronic kidney disease (CKD) stage. Secondary outcomes included LS changes measured via TE and ARFI. Additional end points included MELD-Na, MELD 3.0, CTP scores, hepatic decompensations, proteinuria, body mass index (BMI), hemoglobin A1c (Hb-A1c), blood glucose (BG).

**Results:**

At baseline, the two groups were comparable in GFR (SGLT2i: 55.6 ± 1.9 vs. insulin: 58.1 ± 2.1 mL/min/1.73 m², p = 0.37), CKD stage, ARFI-SWV (2.9 ± 0.1 vs. 2.8 ± 0.1 m/s, p = 0.26), MELD-Na, and MELD 3.0. The SGLT2i group was older (p < 0.01) and had higher AST (p=0.01), ALT (p<0.01), and CTP scores (p=0.02), but lower LS by TE (p = 0.03). Over 48 months, GFR increased in the SGLT2i group (+13.5 ± 1.3) and declined in the insulin group (−4.2 ± 1.4; p < 0.01). A greater proportion of SGLT2i patients transitioned from CKD stage 3a to 2 (p = 0.04). Liver stiffness by TE decreased in the SGLT2i group (−4.0 ± 1.1 kPa), while it increased in the insulin group (+3.0 ± 2.5 kPa; p < 0.01). ARFI-SWV also declined in the SGLT2i group but increased in the insulin group (2.5 ± 0.1 vs. 3.2 ± 0.1 m/s; p < 0.01). The SGLT2i group also demonstrated significant improvement in MELD-Na, MELD 3.0 and CTP scores, with greater resolution of hepatic decompensations, proteinuria, as well as better BMI and HbA1c outcomes (all p < 0.01).

**Conclusions:**

Patients with CTP B cirrhosis and T2DM receiving SGLT2i therapy experienced a significant improvement in renal, hepatic function, and glycemic control over 48 months compared to patients treated with insulin.

## Introduction

Renal dysfunction is a common and difficult to manage comorbidity in patients with cirrhosis. Chronic kidney disease (CKD) predisposes patients with cirrhosis to worse renal outcomes, as well as reduced overall survival (1). Among patients with advanced liver disease, metabolic dysfunction-associated steatotic liver disease (MASLD) is significantly associated with an increased incidence and prevalence of CKD (2–4). Type 2 diabetes mellitus (T2DM) is a prevalent metabolic comorbidity in MASLD and acts as an independent risk factor for CKD (5); in fact, renal dysfunction occurs more frequently in diabetic compared with non-diabetic patients with MASLD (6). Furthermore, patients with cirrhosis exhibit reduced hepatic insulin clearance and increased diversion of insulin from the portal to the systemic circulation (7), thus contributing to insulin resistance and the progression of MASLD.

T2DM and MASLD are two closely intertwined disease entities, with about 70% of patients with T2DM having MASLD globally (8). Thus, there is a clear need for a multimodal therapeutic agent that can prevent the progression of CKD in patients with cirrhosis secondary to MASLD. In this context, sodium-glucose cotransporter-2 inhibitors (SGLT2i) have emerged as a promising therapeutic option, potentially offering both glycemic control and renal protection (9).

SGLT2 inhibitors are now recognized as safe, disease-modifying therapy for T2DM, atherosclerotic cardiovascular disease, heart failure (HF) and CKD (10). While initially developed for glycemic control, their benefits extend beyond diabetes, including improvements in cardiorenal outcomes (11–13). In each of these disease states, SGLT2i have been associated with a significant nephroprotective effect. Several mechanisms have been proposed for their nephroprotective effects, including modulation of the renin-angiotensin-aldosterone system (RAAS), reduction of intraglomerular pressure, and suppression of inflammatory mediators such as interleukin-6, nuclear factor-kB (14).

Despite the broadening clinical use of SGLT2is, their impact in patients with chronic liver disease remains underexplored. Emerging data suggest that SGLT2i may reduce hepatic fat content and fibrosis in MASLD (15, 16), with additional case reports describing benefit in individuals with cirrhosis and refractory ascites (17). Given that diabetes independently increases the risk of major complications in cirrhosis (18, 19), the study of SGLT2is in patients with MASLD cirrhosis and T2DM is of particular relevance.

For this reason, we aimed to assess the longitudinal effect of SGLT2i therapy on renal function, hepatic outcomes and glycemic control in patients with Child-Turcotte-Pugh (CTP) B cirrhosis and T2DM.

## Materials and methods

### Study design and patient selection

We conducted a longitudinal, retrospective cohort study of patients with Child-Turcotte-Pugh B cirrhosis secondary to metabolic dysfunction-associated steatotic liver disease (MASLD) and comorbid type 2 diabetes mellitus (T2DM). Patients were identified via electronic health record review using diagnostic keywords including “cirrhosis,” “Child-Turcotte-Pugh B,” “steatohepatitis,” “MASLD,” “type 2 diabetes mellitus,” “SGLT2 inhibitor,” and “insulin,” in combination with medication records.

Eligible patients were required to have at least 48 months of continuous follow-up. Data collection began in January 2019, with the last eligible inclusion in April 2020, permitting up to 48 months of follow-up through April 2024 depending on the date of enrollment. No patients were lost to follow-up during the 48-month study period.

Inclusion criteria also required serial renal function assessments and serial measurements of liver stiffness (LS). Patients in the SGLT2i group received a single SGLT2 inhibitor (canagliflozin, dapagliflozin, or empagliflozin) as their sole antihyperglycemic agent throughout the study. Patients receiving additional glucose-lowering therapies or with alternate etiologies of liver disease were excluded. Importantly, none of the study participants consumed a clinically significant amount of alcohol. Furthermore, none of the patients were taking medications known to directly affect liver stiffness or induce weight loss during the study period.

Insulin was selected as the comparator due to its long-standing clinical use and safety profile in patients with decompensated cirrhosis (20, 21).

Of note, the diagnosis of cirrhosis was performed using a combination of clinical evaluation, laboratory findings, imaging, and, when applicable, ascitic fluid analysis and liver stiffness measurement via transient elastography (TE) or acoustic radiation force impulse (ARFI). Diagnostic criteria were based on the American Association for the Study of Liver Diseases (AASLD) guidelines (22). Specifically, cirrhosis was diagnosed in the presence of physical stigmata of chronic liver disease, persistently abnormal liver function tests, ultrasonographic features suggestive of cirrhosis and/or portal hypertension, a serum-ascites albumin gradient (SAAG) ≥1.1 g/dL with ascitic fluid protein <2.5 g/dL, and elevated liver stiffness values (22). Liver biopsy was not performed due to procedural risks and the availability of validated non-invasive alternatives.

Treatment group assignment (SGLT2i vs. insulin) reflected individualized clinical decisions made by providers within standard-of-care guidelines. Insulin was typically selected when tighter glycemic control was deemed necessary or when providers preferred a familiar and titratable therapy, particularly in the context of cirrhosis. SGLT2 inhibitors were prescribed in patients considered appropriate candidates, especially when potential cardiorenal benefits were prioritized. Combination therapy with both insulin and an SGLT2 inhibitor was rare in this population and was therefore excluded from analysis; including such patients would not have allowed for meaningful statistical comparison and could have compromised interpretability of the results.

### Data collection

Demographic, clinical, laboratory, and outcome data were extracted from subjects’ medical records. Demographic data included age and biologic sex. Key clinical characteristics were recorded: diabetes duration, medical comorbidities, weight, body mass index (BMI), presence of ascites, hepatic encephalopathy, esophageal varices, occurrence of esophageal variceal bleeding.

Laboratory data was collected every 3 months and included: serum creatinine, glomerular filtration rate (GFR), hemoglobin-A1c (Hb-A1c), blood glucose (BG), hemoglobin (Hb), serum sodium, alanine aminotransferase (ALT), aspartate aminotransferase (AST), alkaline phosphatase (ALP), total bilirubin, platelet count, and international normalized ratio (INR), and urine protein level. N-terminal-pro-brain natriuretic peptide (NT-proBNP) was also longitudinally collected.

The following liver disease severity and fibrosis indices were calculated and reported: Model for End-Stage Liver Disease-Sodium (MELD-Na), MELD 3.0, Child-Turcotte Pugh (CTP) and Fibrosis-4 (FIB-4) index scores.

Liver stiffness (LS) and controlled attenuation parameter (CAP) were measured every 6 months in the patients without ascites via transient elastography (TE). Acoustic radiation force impulse with shear wave velocity quantification (ARFI-SWV) was used for all patients, including individuals with ascites, to assess liver stiffness. Surveillance esophagogastroduodenoscopy, abdominal ultrasound and echocardiographic assessment were also performed as part of routine clinical care.

A longitudinal summary of medication use in both treatment groups was also collected. Diuretic use was categorized by specific drug type (potassium canrenoate and/or furosemide) and reported at baseline and at the end of the study (48 months); new initiation and discontinuation of diuretics during the study were also reported. Similarly, the use of beta-blockers, angiotensin-converting enzyme inhibitors (ACEi), and angiotensin receptor blockers (ARBs) was also recorded. Total daily insulin use (in both U/kg/day and U/day) as well as daily basal and rapid-acting insulin doses were also tracked over the course of the study period.

For patients in the insulin-treated cohort, detailed insulin dosing data were collected at baseline and again at 48 months. This included total daily insulin dose (in both units/day and units/kg/day), as well as separate documentation of basal and rapid-acting insulin components. Glargine Toujeo was used as the basal insulin, and Lispro Humalog was the rapid-acting formulation administered. Adherence was routinely reinforced at each 3-month follow-up visit, and no concerns regarding compliance were documented.

### Fibrosis assessment

LS was assessed by TE (FibroScan; Echosens, Paris, France). TE was performed as reported by Sandrin et al. (23), using at least 10 valid measurements; examinations were considered reliable when interquartile range was <30% and the success rate was >60%. To define the presence of significant fibrosis, we used the cut-off value of 7.9 kPa, as proposed by others (24). Moreover, we measured CAP by measuring the attenuation at the center frequency of the FibroScan (25), ensuring that the liver ultrasonic attenuation was obtained simultaneously from the same volume of liver parenchyma as that of LS. CAP values range from 100 to 400 dB/m: the cut-off values we chose to indicate steatosis as absent, mild, moderate and severe were <236, ≥236, ≥270, and ≥302, respectively (26).

Transient elastography was only performed in patients without ascites. For patients with ultrasonographic evidence of ascites, transient elastography was not conducted due to known limitations in accuracy and feasibility in this setting. Instead, acoustic radiation force impulse elastography with shear wave velocity (ARFI-SWV) quantification was employed in all patients for the assessment of liver stiffness. ARFI is not affected by the presence of ascites and has been reliably performed in both patients with and without ascites (27, 28).

### Clinically significant portal hypertension

Clinically significant portal hypertension (CSPH) was assessed using the Baveno VII consensus criteria (29). In patients without ascites, LS measurement by transient elastography was used to determine CSPH status. A LS ≥25 kPa was considered sufficient to rule in CSPH, while a LS ≤15 kPa combined with a platelet count ≥150×10^9^/L was used to rule out CSPH. For LS values between 20–25 kPa, CSPH risk was further stratified using platelet count, wherein values <150×10^9^/L indicate ≥60% likelihood of CSPH. In patients with ascites, TE was not feasible; ARFI-SWV was employed instead. Prior studies have shown that an ARFI-SWV cutoff ≥ 2.58 m/s correlates with a hepatic venous pressure gradient (HVPG) ≥10 mmHg, thereby identifying CSPH in this setting (27, 30). CSPH classification was determined at baseline and again at 48 months for all patients.

### Study end points

The primary aim of the study was to assess the effect of SGLT2i therapy on renal function compared to insulin. The primary outcome was assessed in two ways: a) change in glomerular filtration rate (GFR) and b) change in chronic kidney disease (CKD) stage over time. Of note, GFR was selected as the primary renal outcome instead of serum creatinine as it provides a more sensitive and direct measure of kidney function, particularly in early renal impairment. GFR was analyzed as a continuous variable to detect subtle treatment effects and reduce potential limitations associated with discrete CKD stage thresholds. To complement this approach, categorical changes in CKD stage were also measured.

The secondary aim was to assess the impact of SGLT2i therapy compared to insulin on liver fibrosis; the outcome measure was assessed by change in LS over time measured both via TE and ARFI.

Additional end points included evaluation of liver disease severity via MELD-Na, MELD 3.0 and CTP scores. The incidence of new onset or resolution of the following conditions was also monitored over time: ascites, varices, hepatic encephalopathy and proteinuria.

Changes in the following clinical and laboratory variables were also monitored over time: BMI, Hb-A1c, BG, Hb, NT-proBNP. The baseline use and the subsequent changes in the following medication classes were also documented: diuretics type, non-selective beta-blockers, angiotensin converting enzyme inhibitors (ACEi) and angiotensin receptor blockers (ARB).

### Safety

Data on both non-serious and serious adverse events were collected throughout the course of the study period.

### Statistical analysis

Demographic, clinical and laboratory data characteristics were compared at baseline using chi-squared, student’s t test or Mann-Whitney U tests, depending on distribution. The primary outcome was evaluated using a linear mixed-effect model fitted with GFR as the dependent variable. The fixed effects included medication type, time, their interaction, along with the following covariates: baseline ascites, baseline albumin and baseline MELD-Na. When the model was run with MELD 3.0 as covariate, albumin was not included in the analysis as this variable is already included in the calculation of MELD 3.0. The model included random intercepts and random slopes for each patient to account for individual variability in both baseline GFR and the rate of change in GFR over time. To evaluate the secondary outcome, a linear mixed model was also fitted for liver stiffness; the fixed effects included medication type, time, their interaction, along with the following covariates: baseline MELD-Na, baseline BMI and change in BMI between the start and end of the study; a similar linear-mixed model for LS was run utilizing MELD 3.0 as well. Covariates in each model were selected *a priori* based on clinical relevance. Data were assessed at p = 0.05 for significance and p = 0.15 was considered a trend. Data was analyzed and graphed using IBM SPSS Statistics (Version 29) and Python programming language 3.12.

This study was approved by the Institutional Review Board (IRB No. 176/18) and was performed in accordance with the ethical standards of the institutional research committee. The need for informed consent was waived because of the retrospective nature of the study. The reporting of this study conforms to the Strengthening the Reporting of Observational Studies in Epidemiology (STROBE) (31).

## Results

### Baseline characteristics

Fifty-four patients were included in the study, of which 27 patients were initiated on SGLT2i therapy and 27 on insulin. The median age was higher in the SGLT2i group (66 years, IQR 60-69) compared to the insulin group (59 years, IQR 57-61) (p <0.01). Patients in the SGLT2i group also had higher BMI (p=0.04), AST (p= 0.01), ALT (p <0.01). Despite having higher CTP scores (9 points, IQR 7–9 vs. 8 points, IQR 7-9, p= 0.02), the SGLT2i group had lower liver stiffness at baseline compared to the insulin group (28.5 kPa ± 0.8 vs. 32.8 kPa ± 1.7, p = 0.03). The median CKD stage was CKD3a and the mean GFR did not differ between the SGLT2i and insulin groups (55.6 ± 1.9 mL/min/1.73 vs 58.1 ± 2.1 mL/min/1.73 m², p= 0.37). There were no significant differences in duration of diabetes, Hb-A1c, BG, MELD-Na and MELD 3.0 scores, presence of CSPH, and frequencies of decompensations between groups. Furthermore, all patients had no evidence of heart failure and the NT-proBNP serum levels were similar across the two groups at baseline. **Table 1** includes the complete baseline characteristics. There was no difference in diuretics, beta-blockers, ACE inhibitors (ACEi), angiotensin receptor blockers (ARBs) use at the beginning of the study across the two groups. A complete longitudinal summary of the medications used in both treatment groups is provided in **Supplementary Table 1**. Furthermore, there were no significant differences in patients’ baseline prevalence of other medical comorbidities (**Supplementary Table 2**).

**Table 1:** Baseline characteristics of the patients are presented in **Table 1**.

Variable	SGLT2 (n = 27)	Insulin (n = 27)	p value (2-sided)
Age (yr)	66 (60,69)	59 (57,61)	<0.01
Sex (female)	12 (44%)	13 (48%)	1.00
T2DM duration (years)	11.6 ± 0.6	10.4 ± 0.5	0.15
Bodyweight (Kg)	74.40 ± 2.0	77.5 ± 1.3	0.30
BP systolic (mmHg)	138.2 ± 1.3	133.8 ± 1.0	0.01
BP diastolic (mmHg)	81.8 ± 1.1	82.6 ± 0.9	0.60
BMI (m/Kg^2^)	28.4 ± 0.2	27.7 4± 0.3	0.04
Hb-A1c (%)	7.9 ± 0.1	7.9± 0.1	0.81
BG (mg/dL)	150.4 ± 2.5	147.9 ± 2.9	0.52
Hb (g/dL)	11.3 ± 0.2	11.0 ± 0.1	0.11
Cr (mg/dL)	1.2 ± 0.1	1.1 ± 0.0	0.31
GFR (mL/min/1.73m^2^)	55.6 ± 1.9	58.1 ± 2.1	0.40
CKD stage	3a (2, 3b)	3a (2, 3b)	0.61
Urine protein	14 (52%)	7 (52%)	0.09
Na (mm/L)	139.8 ± 0.4	139.4 ± 0.25	0.43
ALT (U/L)	47.3 ± 1.1	42.3 ± 0.9	<0.01
AST (U/L)	53.1 ± 2.1	46.5 ± 1.0	0.01
Platelets (cells/mm^3^)	129.9 ± 2.6	128.0 ± 1.6	0.55
Albumin (g/dL)	2.9 ± 0.1	3.0 ± 0.0	0.37
Bilirubin (mg/dL)	1.1 ± 0.1	2.5 ± 0.1	0.18
INR	1.2 ± 0.0	1.1± 0.1	0.62
CSPH	27 (100%)	27 (100%)	1.00
MELD-Na	13.1 ± 0.2	12.6 ± 0.2	0.10
MELD 3.0	14.7 ± 0.2	14.3 ± 0.3	0.18
Child-Pugh class	9 (7, 9)	8 (7, 9)	0.02
Fib4	3.4 ± 0.3	3.2 ± 0.1	0.60
Ascites	20 (74%)	17 (63%)	0.55
Varices	21 (78%)	26 (96%)	0.10
Variceal bleeding	0	0	NA
Hepatic encephalopathy	18 (67%)	10 (37%)	0.06
Ejection Fraction (%)	61 ± 1	60 ± 1	0.49
CAP (dB/m)	301.4 ± 6.7	330.8 ± 26.7	0.01
LS (kPa)	28.5 ± 0.8	32.8 ± 1.7	0.03
ARFI-SWV (m/s)	2.9 ± 0.1	2.8 ± 0.1	0.26
NT-proBNP (pg/mL)	196.6 ± 8.5	184.3 ± 8.9	0.32

A p-value of 0.05 or less was considered statistically significant. Of note, LS and CAP measurement are only provided for patients without ascites at the beginning of the study.

### Primary outcome

**Figure 1:**
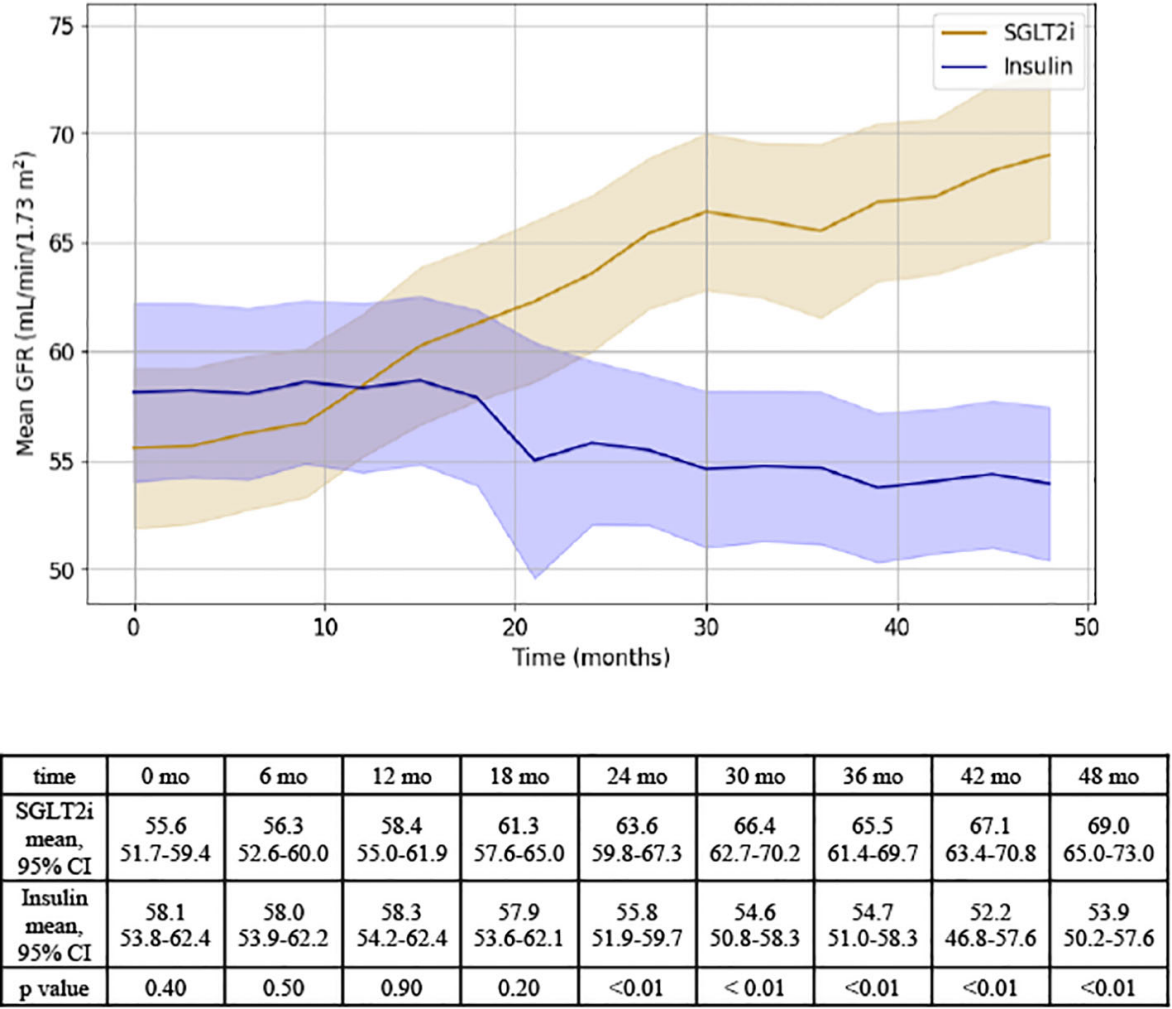
Representation of GFR changes over time for SGLT2i and insulin groups. Results of independent T test analysis comparing the mean GFR at 6 month intervals for the SGLT2i and insulin groups are provided.

After 48 months, patients prescribed an SGLT2i experienced a 13.5 ± 1.3 point improvement in GFR while patients initiated on insulin therapy experienced a - 4.2 ± 1.4 point downtrend in GFR (**Figure 1**).

On mixed linear regression modeling, the following variables were associated with GFR: medication (F=10.5, p < 0.01), time (F=6.0, p< 0.01), and MELD-Na (F=5.2, p <0.01). Medication type was associated with a significantly different effect on GFR (F= 10.5, p < 0.01); in addition, a significant interaction was detected between medication group and time (F = 31.5, p < 0.01), supporting that there was both a difference in the effect of each medication on GFR and that there was a difference in the change in GFR over time between groups. Baseline ascites did not have a significant effect on GFR, yet there was a trend towards significance for baseline albumin (**Supplementary Table 3a**).

When MELD 3.0 was used in place of MELD-Na, results remained consistent: medication (F = 35.4, p < 0.001), time (F = 18.1, p < 0.001), and the medication*time interaction (F = 30.0, p < 0.001) were all significantly associated with GFR. The effect of baseline MELD 3.0 was not statistically significant (F = 1.6, p = 0.14), and baseline ascites remained non-significant (F = 0.04, p = 0.85) (**Supplementary Table 3b**).

**Figure 2:**
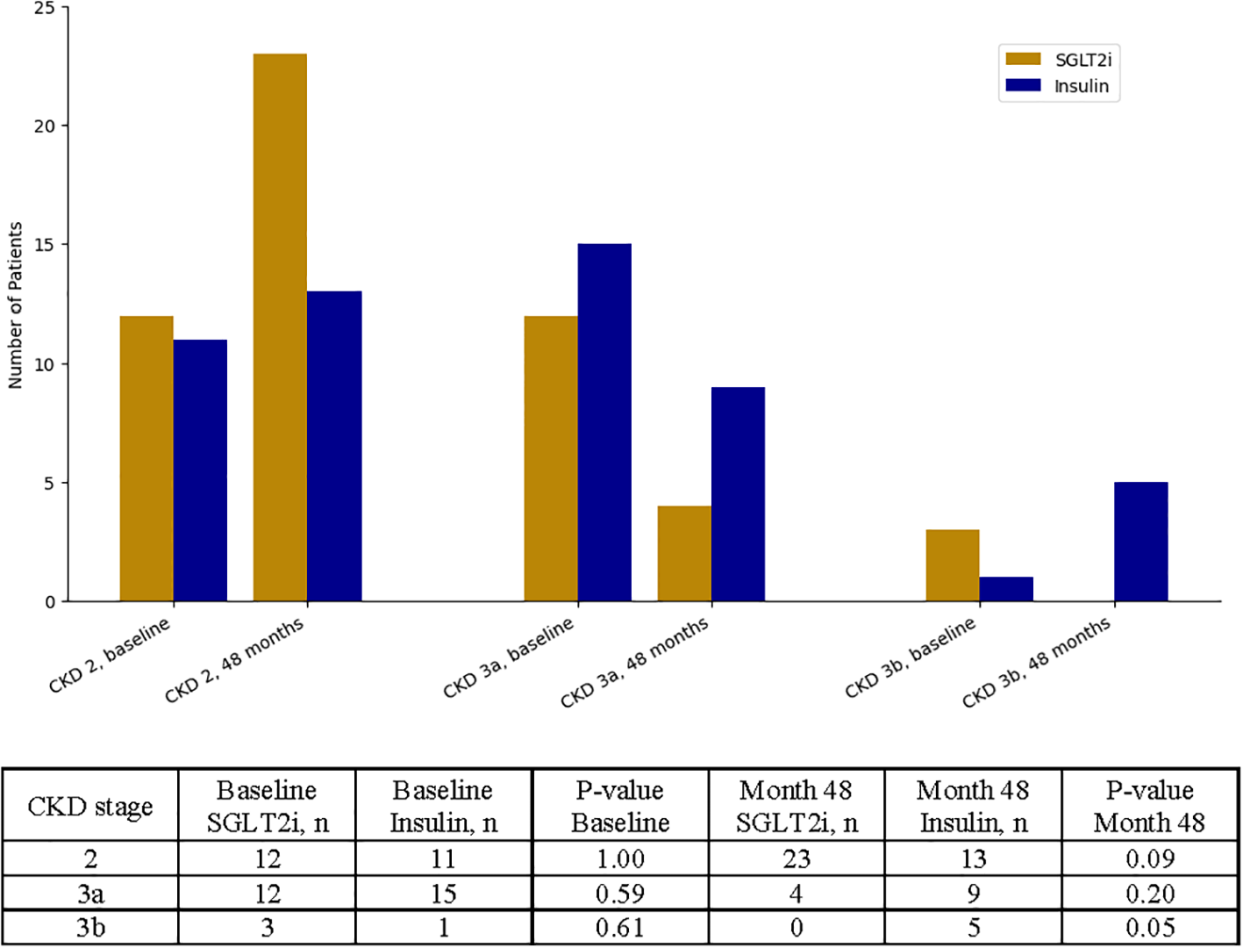
Categorical analysis of chronic kidney disease (CKD) stage progression over time. Patients were stratified by CKD stage (stage 2, stage 3a, and stage 3b) at baseline and at 48 months. Counts and corresponding p-values for between-group comparisons at each time point are shown.

To complement the primary outcome analysis of GFR as a continuous variable, we also evaluated renal function categorically by measuring changes in CKD stage. At baseline, the distribution of CKD stages was similar between the SGLT2i and insulin groups. However, by month 48, a significantly greater proportion of patients in the SGLT2i group experienced an improvement in CKD stage compared to the insulin group (12 vs. 2 patients, p < 0.01). Patients treated with SGLT2 inhibitors were more likely to be classified as CKD stage 2, whereas patients in the insulin group had a higher proportion of individuals in CKD stage 3a or 3b (**Figure 2**).

Notably, a significantly greater number of patients treated with SGLT2i transitioned from CKD stage 3a to stage 2 compared to those in the insulin group (p = 0.04), suggesting a meaningful improvement in renal function over time. Detailed transition patterns between CKD stages are reported in **Supplementary Table 4**.

### Secondary outcome

**Figure 3:**
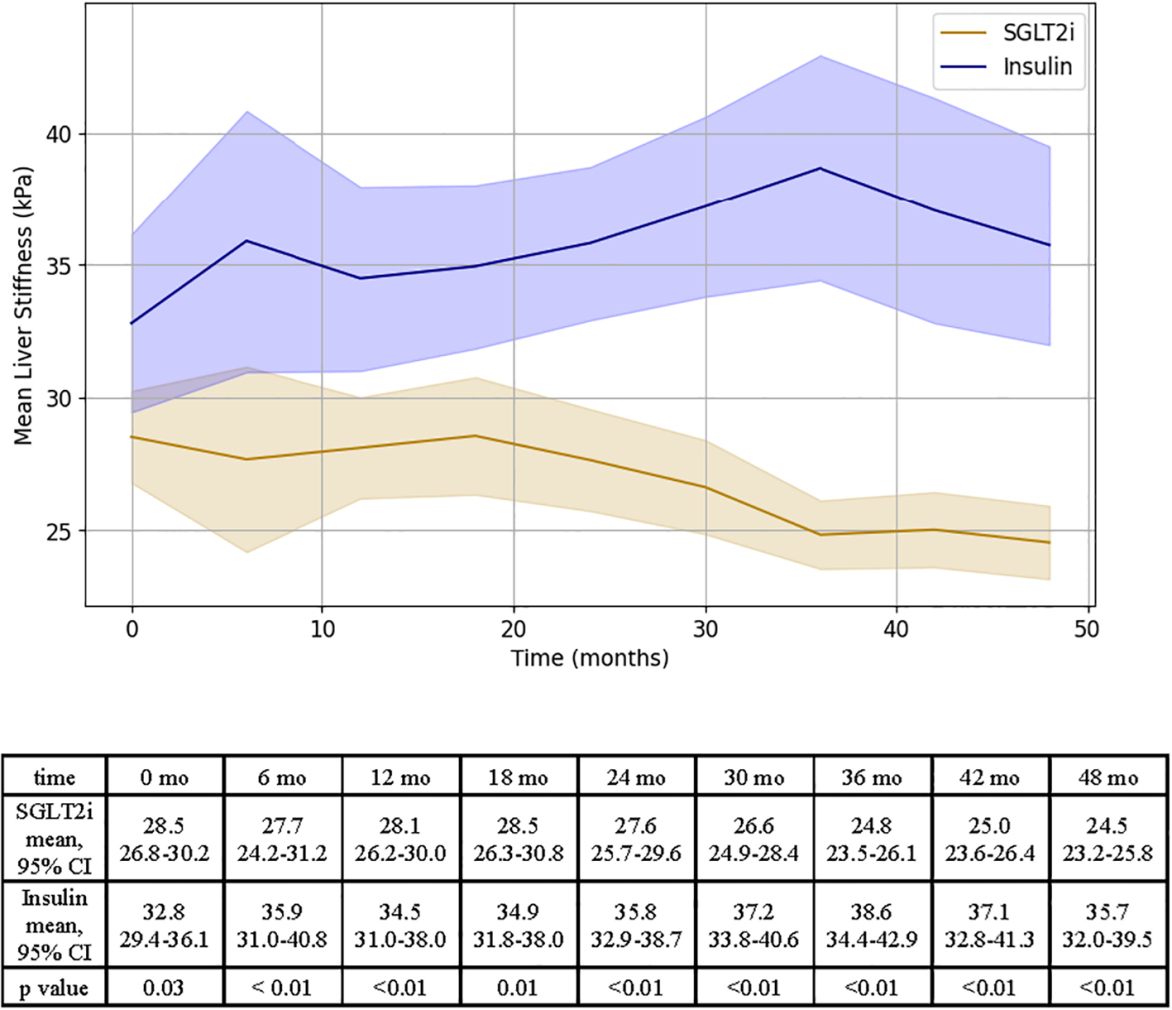
Representation of LS changes over time for SGLT2i and insulin groups. Results of independent T test analysis comparing the mean LS at 6 month intervals for the SGLT2i and insulin groups are provided.

After 48 months, patients prescribed an SGLT2i experienced a decrease in liver stiffness of 4.0 ± 1.1 kPa, while patients in the insulin group experienced a +3.0 ± 2.5 kPa increase in LS (p < 0.01, see **Figure 3**).

The MELDNa-based mixed linear regression model for LS showed that the following variables were associated with liver stiffness: medication (F= 15.7, p < 0.01), time (F=2.0, p=0.04, **Supplementary Table 5a**). This association remained statistically significant after adjusting for change in BMI between the start and end of the study period, however an attenuation in the effect of medication on LS was observed (F =20.3, p=0.05, **Supplementary Table 5b**).

When MELD 3.0 was used in place of MELDNa, the medication effect on LS remained statistically significant prior to introducing change in BMI (**Supplementary Table 5c**). Once the change in BMI over the length of the study period was accounted for, the medication effect was attenuated and no longer statistically significant (F = 14.6, p = 0.16, **Supplementary Table 5d**).

In all models, the interaction between medication and time remained highly significant (p <0.01, **Supplementary Tables 5a-d**), supporting that there was both a difference in the effect of medication type on LS and that there was a difference in the change in LS over time between groups.

Consistent with the TE findings, ARFI-SWV significantly decreased in the SGLT2i group over the 48-month period (2.9 ± 0.1 m/s at baseline vs. 2.5 ± 0.1 m/s at 48 months, p < 0.01, **Table 2**), while ARFI-SWV significantly increased in the insulin group (2.8 ± 0.1 m/s at baseline vs. 3.2 ± 0.1 m/s at 48 months, p < 0.01, **Table 2**). Between-group differences at 48 months were also statistically significant (p < 0.01, **Table 2**), further supporting that SGLT2i therapy was associated with favorable changes in liver stiffness.

**Table 2:** Summary of liver stiffness (LS) by TE (kPa) or by ARFI -SWV (m/s) at the start and the end of the study. Mean CAP values at the beginning and end of the study are also included.

Variable	SGLT2i (mean ± se)	p SGLT2i (0 vs 48)	Insulin (mean ± se)	p Insulin (0 vs 48)	p (SGLT2i vs Insulin)
LS_0_ (kPa)	28.5 ± 0.8		32.8 ± 1.7		0.03
LS_48_ (kPa)	24.5 ± 0.7	<0.01	35.7 ± 1.9	0.02	<0.01
ARFI-SWV_0_ (m/s)	2.9 ± 0.1		2.8 ± 0.1		0.26
ARFI-SWV_48_ (m/s)	2.5 ± 0.1	<0.01	3.2 ± 0.1	<0.01	<0.01
CAP_0_ (dB/m)	301.4 ± 6.7		330.8 ± 26.7		0.01
CAP_48_ (dB/m)	266.9 ± 3.4	<0.01	323.4 ± 13.1	0.63	<0.01

Furthermore, as shown in **Table 2**, patients in the SGLT2i group also experienced a significant reduction in CAP scores over the 48-month study period (301.4 ± 6.7 dB/m at baseline to 266.9 ± 3.4 dB/m at study end, p < 0.01). In contrast, CAP scores in the insulin group did not significantly change during the study (330.8 ± 26.7 dB/m to 323.4 ± 13.1 dB/m, p = 0.63).

Clinically significant portal hypertension status was also assessed at study completion. At baseline, all patients met the criteria for CSPH. By month 48, all patients treated with insulin continued to meet CSPH thresholds, either through TE-based LSM ≥25 kPa (mean 35.7 ± 1.9) or persistently elevated ARFI-SWV (mean 3.2 ± 0.1 m/s). In contrast, the majority of SGLT2i-treated patients had LS values <25 kPa at study end (mean 24.5 ± 0.7), yet all had platelet counts <150×10^9^/L (mean 134.0 ± 0.6). According to Baveno VII criteria, this combination confers a ≥60% likelihood of CSPH, suggesting that while liver stiffness improved significantly in the SGLT2i group, some patients may have continued to harbor CSPH at study completion.

### Other end points

**Figure 4:**
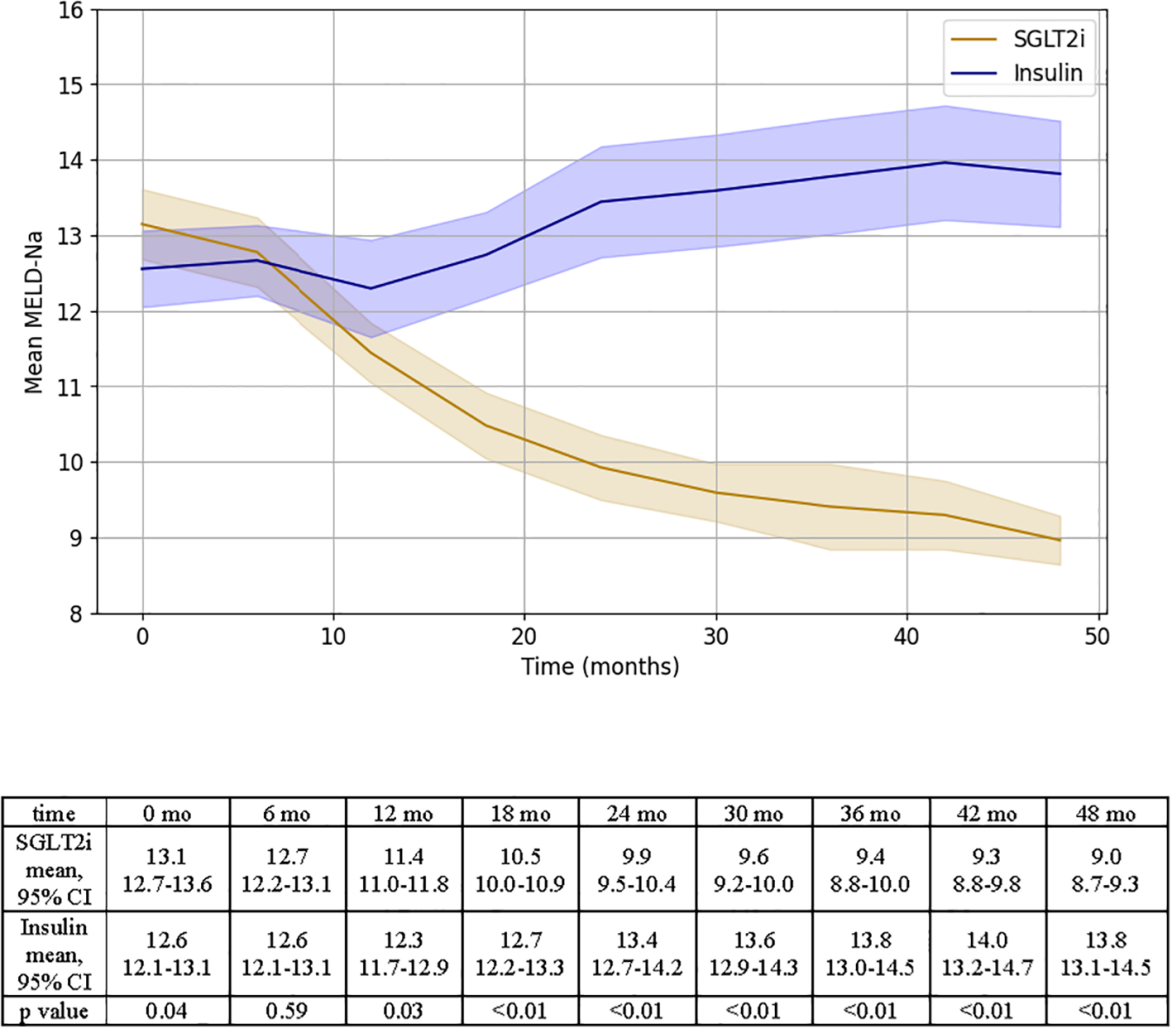
Representation of MELD-Na changes over time for SGLT2i and insulin groups. Results of independent T test analysis comparing the mean MELD-Na at 6 month intervals for the two groups are provided.

The mean MELD-Na was significantly lower at the end of the study for patients in the SGLT2i group (MELD-Na_end_ 9.0 ± 0.3 vs. MELD-Na_start_ 13.1 ± 0.2, p< 0.01). Instead, patients on insulin experienced an increase in MELD-Na throughout the study (MELD-Na_end_ 13.8 ± 0.3 vs MELD-Na_start_ 12.6 ± 0.2, p<0.01, **Figure 4**). MELD 3.0 scores were also assessed at 6-month intervals and displayed a consistent trend with MELD-Na (**Supplementary Figure 1**). Specifically, patients in the SGLT2i group showed a progressive and statistically significant decline in MELD 3.0 score from 14.7 ± 0.3 at baseline to 10.2 ± 0.2 at 48 months (p < 0.01). Conversely, MELD 3.0 scores worsened among patients treated with insulin, increasing from 14.3 ± 0.3 to 15.6 ± 0.4 over the same period (p < 0.01).

**Figure 5:**
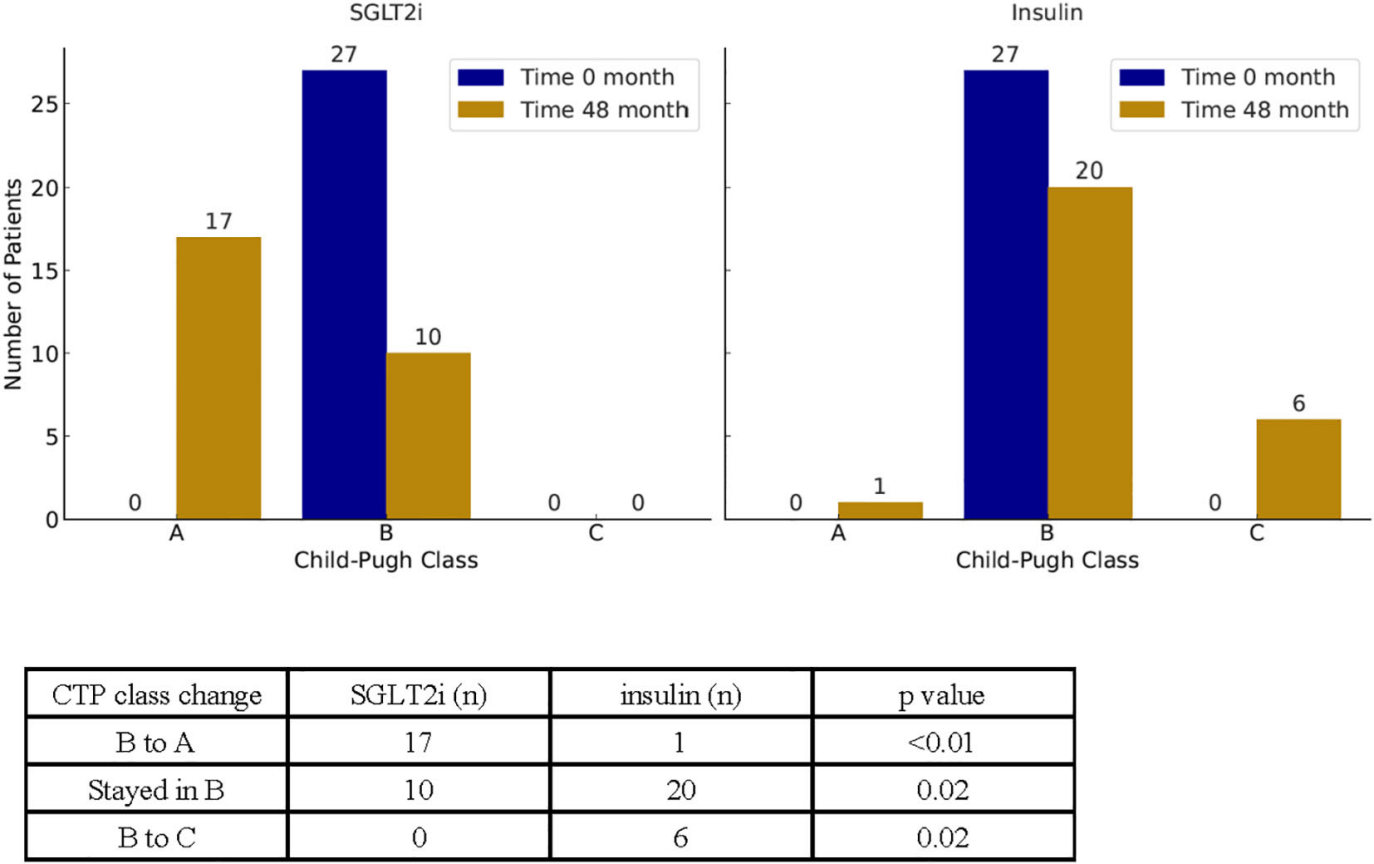
Illustration of the changes in CTP class between the start and end of the study for SGLT2i and insulin groups. Counts of CTP class changes over time are reported in the table below.

In parallel with changes in MELD-based scoring systems, we also examined changes in CTP class over the 48-month study period (**Figure 5**). At baseline, all patients were classified as Child-Pugh B. By the end of the study, 17 of 27 patients (63%) in the SGLT2i group improved to CTP A, compared to only 1 of 27 (3.7%) in the insulin group (p < 0.01). Additionally, 6 patients (22%) in the insulin group progressed from CTP B to C, whereas no patients in the SGLT2i group experienced progression to class C (p = 0.02).

To better understand the drivers of the changes observed in the MELD and CTP scores over time, we conducted a component-level analysis of each scoring system (**Supplementary Table 6**). In the SGLT2i group, significant reductions were observed in serum bilirubin (−0.5 ± 0.1 mg/dL, p < 0.01), serum creatinine (−0.2 ± 0.0 mg/dL, p < 0.01), and INR (−0.2 ± 0.0, p < 0.01), along with an increase in serum albumin (+0.3 ± 0.0 g/dL, p < 0.01). These changes contributed meaningfully to the observed reduction in MELD-Na and MELD 3.0 scores over time (**Supplementary Table 6a**). In contrast, the insulin group showed either no improvement or worsening in these components (**Supplementary Table 6a**). For the CTP score, the most notable between-group differences were observed in ascites, hepatic encephalopathy, bilirubin, and albumin subcomponents. All showed statistically significant improvements in the SGLT2i group compared to the insulin group (**Supplementary Table 6b**).

**Figure 6:**
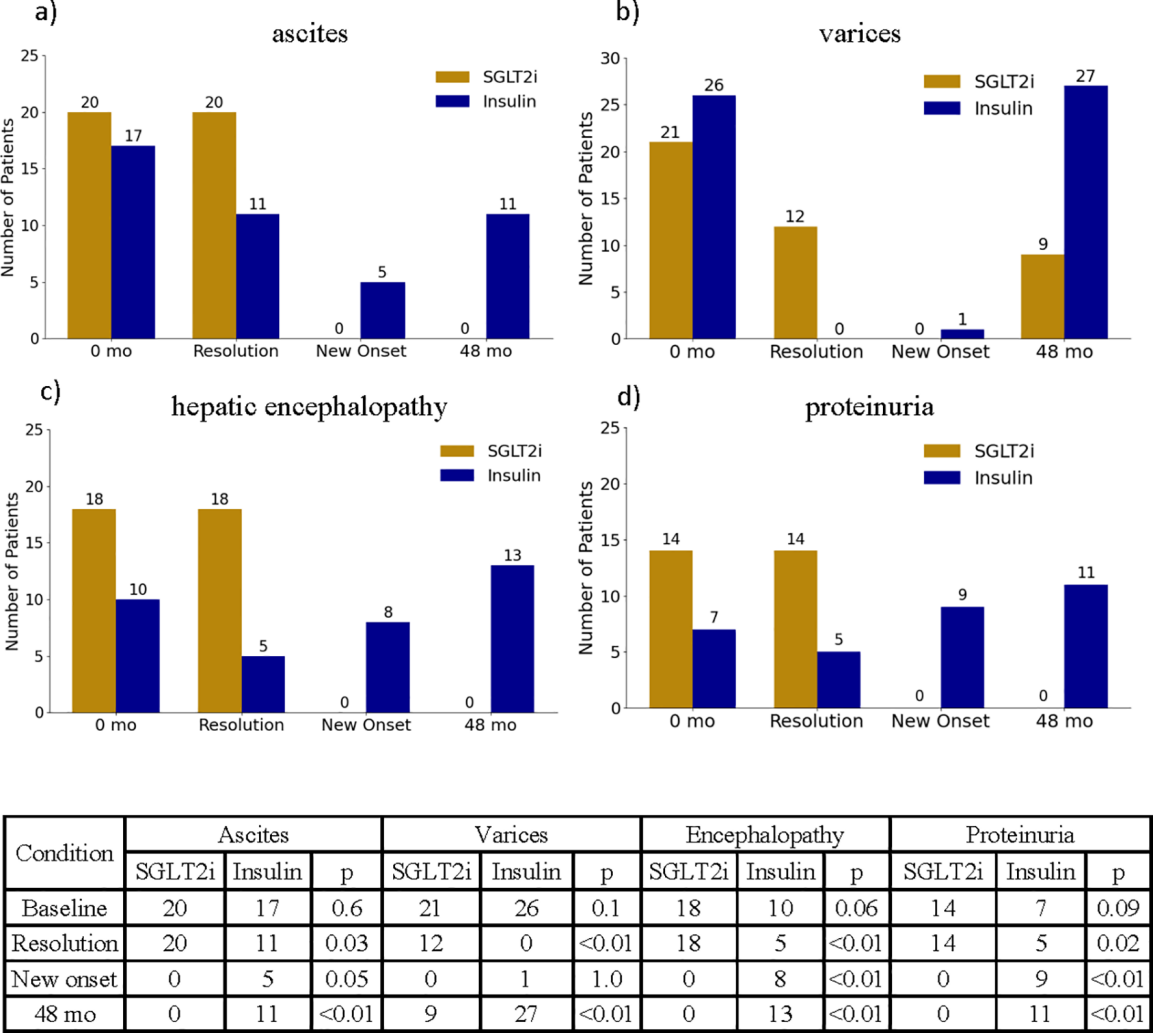
Illustration of the changes in ascites **(a)**, varices **(b)**, hepatic encephalopathy **(c)** and proteinuria **(d)** between start and end of study period. Counts of ascites, varices, hepatic encephalopathy and proteinuria at baseline and end of the study period, as well as new onset and resolution of these conditions are summarized in the table below.

The prevalence of hepatic decompensations also differed at the end of the study period between treatment groups (**Figure 6**). Among patients with baseline ascites, 20 of 20 patients in the SGLT2i group experienced complete resolution of ascites by 48 months compared to 11 of 17 patients in the insulin group (p = 0.03). New onset ascites occurred in 0 patients in the SGLT2i group versus 5 patients in the insulin group (p=0.05, **Figure 6a**). Similarly, among patients with baseline varices, 12 of 21 patients in the SGLTi group had resolution of varices on follow up endoscopy, whereas none of the 26 patients in the insulin group showed variceal resolution (p<0.01, **Figure 6b**). Of note, only one patient in the SGLT2i group experienced an episode of esophageal bleeding; the event was successfully managed via endoscopic band ligation, and SGLT2i therapy was resumed at hospital discharge and continued without interruptions for the remainder of the study. The incidence of hepatic encephalopathy followed a similar pattern. Of the 18 patients with baseline encephalopathy in the insulin group, only 5 showed resolution, compared to 10 of 10 in the SGLT2i group (p < 0.01, **Figure 6c**). New onset encephalopathy was more common in the insulin group (p = 0.04).

Resolution of proteinuria was also significantly more frequent in the SGLT2i group, with all 14 patients with baseline proteinuria demonstrating complete resolution by the end of the study (**Figure 6d**).

Furthermore, 9 patients in the insulin treated group experienced new onset of proteinuria, while this phenomenon occurred in 0 patient in the SGLT2i group (p <0.01, **Figure 6d**).

By the end of the study period, the SGLT2i group also exhibited significantly lower NT-proBNP levels compared to the insulin group (138.7 ± 7.2 vs. 194.5 ± 11.4 pg/mL, p < 0.01). Moreover, the change in NT-proBNP over time differed significantly between groups, with a decline observed in the SGLT2i cohort (−57.9 ± 5.7 pg/mL) and an increase in the insulin group (+10.2 ± 5.2 pg/mL).

Changes in BMI and Hb-A1c over time for the SGLT2i and insulin groups were also measured; the SGLT2i group experienced a significant improvement in both these measures compared to the insulin group (p<0.01, **Supplementary Figures 2**, **3**). To assess the validity of Hb-A1c values, Hb and BG were also measured; the change in Hb did not significantly differ across the two groups over time (0.00 vs 0.01, p = 0.92, **Supplementary Figure 4**), while the SGLT2i group experienced a greater reduction in BG compared to the insulin group (26 vs 18 points, p = 0.04, **Supplementary Figure 5**).

### Medication use

Insulin dosing also remained stable over the 48-month study period. Total daily insulin use was 0.49 ± 0.02 U/kg/day at baseline and 0.51 ± 0.01 U/kg/day at 48 months (p = 0.06), and total units/day remained unchanged (38.0 ± 1.4 vs. 38.8 ± 1.0, p = 0.49). Basal insulin use increased (19.1 ± 1.5 to 20.5 ± 1.3 U/day, p = 0.14) while rapid-acting insulin decreased (18.9 ± 0.6 to 18.3 ± 0.7 U/day, p = 0.48), yet neither change reached statistical significance. Full data are provided in **Supplementary Table 7**.

Regarding other medications, 24 patients in the SGLT2i group and 22 in the insulin group were on diuretics at baseline, most commonly a combination of furosemide and potassium canrenoate. by the end of the study, only 9 patients in the SGLT2i group remained on diuretics, compared to 25 in the insulin group (p < 0.01). Beta-blocker use was similar at baseline (21/27 in both groups; p = 1.00) but differed significantly at 48 months (10 vs. 21 patients, p < 0.01). Use of ACE inhibitors and ARBs did not significantly differ between groups at any time point. Full longitudinal data on medication use are presented in **Supplementary Table 1**.

### Adverse events

No major adverse events were reported in either treatment group throughout the 48-month study period. Specifically, there were no documented episodes of euglycemic diabetic ketoacidosis, hypotension, or acute kidney injury. Mild genitourinary tract infections were observed in both groups: in the SGLT2i cohort, 3 patients (11.1%) developed vaginal candidiasis and 1 patient (3.7%) experienced a urinary tract infection (UTI); in the insulin group, 4 patients (14.8%) developed UTIs and 1 patient (3.7%) had vaginal candidiasis. These events were self-limited and did not result in treatment discontinuation.

## Discussion

In our study, patients treated with an SGLT2i exhibited a significant improvement in renal function over 48 months as well as liver stiffness compared to those treated with insulin. The mean change in GFR for the SGLT2i group was +13.5 ± 1.3 mL/min/1.73 m², while the insulin group experienced a decline of -4.2 ± 1.4 mL/min/1.73 m². Furthermore, over the course of the study period, a significantly greater proportion of patients in the SGLT2i group experienced an improvement in CKD stage compared to the insulin group. This represents the first study reporting the nephroprotective effects of SGLT2is in patients with CTP B cirrhosis and T2DM.

Previous studies showed that SGLT2is are beneficial for renal protection in other disease states like heart failure and CKD, irrespective of T2DM status. The CREDENCE trial first showed that the of use the SGLT2i canagliflozin in patients with T2DM and CKD resulted in a significantly lower reduction in GFR over 36 months compared to placebo, as well as reduced onset of end-stage kidney disease (32). Results from the DAPA-CKD trial extended the findings from CREDENCE to patients without T2DM, and showed a significant attenuation in the reduction of GFR also in patients with baseline CKD 4 (11). The findings of these studies are particularly relevant to patients with metabolic liver disease and concomitant T2DM, as they are at increased risk of CKD progression (3, 4). Our findings effectively illustrate the nephroprotective action of using SGLT2is in patients with decompensated cirrhosis secondary to MASLD and T2DM, while also showing their safety in patients with baseline CKD. Importantly, no serious adverse events related to SGLT2i therapy were observed over the 48-month study period. Notably, patients in the SGLT2i group were older and had higher baseline BMI—factors typically associated with worse renal and hepatic outcomes in cirrhosis and T2DM. Despite these less favorable baseline characteristics, the SGLT2i group experienced significant clinical improvement, suggesting that the observed treatment effects may be conservative estimates.

Prior studies have investigated the use of SGLT2is in patients with T2DM and non-alcoholic fatty liver disease (NAFLD). Arai et al. evaluated the antifibrotic effect of SGLT2is in patients with NAFLD and T2DM using the FIB‐4 index as non-invasive assessment of liver fibrosis; patients with intermediate and high risks of advanced fibrosis experienced significant reduction in FIB‐4 index from baseline and this effect was maintained over three years of treatment (33). Several meta-analyses have showed that the use of SGLT2is in patients with NAFLD leads to both a reduction in liver fat content detected by magnetic resonance imaging as well as a small but significant reduction in LS measured by transient elastography (15, 34). Additionally, a clinical investigation by Takeshita et al. on the effects of SGLT-2is in patients with T2DM and NAFLD reported histological scores improvement in liver fibrosis (35). As the use of SGLT2is is been associated with significant weight loss, it is uncertain whether the effects of SGLT2is on hepatic fibrosis and steatosis are due to a direct effect of this medication class or to improvements in other metabolic parameters (36). Our data shows that SGLT2i therapy was associated with a significant reduction in liver stiffness over time both when measured via transient elastography and acoustic radiation force impulse. Accounting for the change in BMI between the start and the end of the study period attenuated the effect of medication on liver stiffness. This indicates that while SGLT2is may have an independent effect of liver fibrosis, this effect is modulated by weight loss.

Emerging experimental and clinical evidence suggests that SGLT2 inhibitors may exert anti-fibrotic and portal pressure-lowering effects beyond their impact on body weight (36, 37). In a diabetic mouse model, Tang et al. demonstrated that dapagliflozin reduced hepatic fibrosis, albuminuria, and glomerulosclerosis, with associated downregulation of fibrotic markers such as TGF-β1, PAI-1, and type IV collagen. Hepatic deposition of fibronectin and types I and III collagen also decreased, alongside with reductions in reactive oxygen species and myeloperoxidase levels, which are key contributors to fibrogenesis and portal hypertension (37). In a prospective human study, Bellanti et al. reported that six months of SGLT2i therapy significantly reduced non-invasive liver fibrosis indices, circulating oxidative stress, and pro-inflammatory cytokines (IL-1β, IL-6, TNF-α), with no such effects observed in patients receiving other hypoglycemic therapies (38). These findings support the hypothesis that SGLT2is modulate hepatic inflammation and fibrosis through mechanisms which are also independent of weight loss and glycemic control.

Despite numerous studies investigating the effect of SGLT2is in patients with NAFLD, there are few studies assessing the efficacy of SGLT2is in patients with cirrhosis. A retrospective study using the TriNetX Research Network identified propensity score-matched patients with T2DM and cirrhosis who were treated with either metformin alone or dual metformin and SGLT2i therapy; the authors showed an associated benefit in reducing both mortality and hepatic decompensations when patients were on dual therapy with both metformin and an SGLT2i versus metformin monotherapy (39). In our study, we show that SGLT2i monotherapy is associated with a significant improvement in hepatic decompensations and that patients treated with a SGLT2i agent experienced significant improvement in MELD-Na, MELD 3.0, and CTP scores, indicating improvement in liver disease severity. Furthermore, while all patients met criteria for clinically significant portal hypertension at baseline, by the end of the study, patients in the SGLT2i group experienced a significant reduction in both liver stiffness by TE (from 28.5 ± 0.8 to 24.5 ± 0.7 kPa) and ARFI-SWV (from 2.9 ± 0.1 to 2.5 ± 0.1 m/s), whereas no improvement was observed in the insulin group. Although all patients in the SGLT2i group continued to have platelet counts <150×10^9^/L - suggesting, per Baveno VII criteria, a residual 60% probability of CSPH (29) - the reduction in liver stiffness measurements supports a potential attenuation of portal pressure over time. Several case reports have also highlighted how SGLT2i therapy alleviates ascites and peripheral edema in patients with decompensated cirrhosis who are refractory to standard diuretic therapy (17, 40). Interestingly, in our study, the need for diuretics decreased significantly in the SGLT2i group over time, with only 9 patients remaining on diuretics at 48 months compared to 25 in the insulin group. This finding highlights the possibility that SGLT2is may reduce ascites through other mechanisms in addition to osmotic diuresis, including antifibrotic and portal pressure-lowering effects.

It is also known that SGLT2is inhibit sodium and glucose reabsorption in the proximal tubule, leading to increased sodium delivery to the macula densa and increased natriuresis. These actions possibly lead to decreased activation of the RAAS and restoration of the tubuloglomerular feedback, with the ultimate effect of decreasing intraglomerular pressure (41, 42). These effects have shown to be nephroprotective over time as they partially prevent renal hyperfiltration. Recent cardiovascular research also showed an important connection between SGLT2 inhibition and attenuation of sympathetic nervous system (SNS) activation (43). As the SNS plays a notable role in sodium retention and renal vasoconstriction in decompensated cirrhosis, SGLT2is may be beneficial at counteracting this vicious pathway. It is possible that these pathways may represent additional avenues through which SGLT2is reduce portal hypertension and lead to improved outcomes.

Finally, our study shows that monotherapy with a SGLT2i agent allows for effective management of T2DM in patients with cirrhosis. It is known that MASLD via mechanisms of increased oxidative stress, inflammation and lipotoxicity worsens insulin resistance (44), and cirrhosis, by decreasing hepatic insulin clearance, also contributes to insulin resistance (7). Despite these limitations, insulin often remains the preferred glycemic controlled agent for patients with decompensated cirrhosis and T2DM. We show that treatment with SGLT2i monotherapy allows for significant improvement in both Hb-A1c and blood glucose measurement over 48 months. This effect is likely due to the ability of SGLT2is to reduce blood glucose independently of insulin sensitivity and secretion, while also promoting peripheral fatty acid oxidation and ketogenesis, which over time reduces pancreatic beta cells stress (45). Importantly, current American Diabetes Association (ADA) guidelines endorse the use of SGLT2 inhibitors as initial therapy for T2DM, without requiring prior metformin use (46, 47). This shift reflects growing recognition of their efficacy and safety as standalone agents. Our findings reinforce the real-world applicability of SGLT2i monotherapy in patients with T2DM and cirrhosis.

Although our longitudinal study underscores the beneficial role of SGLT2i therapy in patients with Child-Pugh B cirrhosis and T2DM, there are several limitations. Our study has a small sample size and only involved two centers. Larger, multi-center studies are needed to further investigate the renal and hepatic effects of SGLT2i therapy in patients with compensated and decompensated cirrhosis irrespective of T2DM status. Such studies would allow to both delineate whether SGLTi therapy can prevent the onset of feared hepatic decompensations as well as improve transplant-free survival.

Overall, our results highlight how treatment with SGLT2 inhibitors can be a valid therapeutic choice to delay and potentially prevent the progression of renal dysfunction and hepatic damage in patients with T2DM and CTP B cirrhosis. To our knowledge, this is the first long-term study to highlight the nephroprotective role of SGLT2i agents in patients with decompensated cirrhosis. Furthermore, treatment with a SGLT2i was associated with a significant improvement in liver stiffness, decrease in MELD-Na and CTP scores, resolution of hepatic decompensations and effective glycemic control. Although larger, multicenter studies are needed to confirm these results, the implications of our findings are significant as they introduce SGLT2is as disease modifying agents in the context of cirrhosis.

## Data Availability

The raw data supporting the conclusions of this article will be made available by the authors, without undue reservation.
