# Supplementary material for: Sodium-glucose cotransporter-2 inhibitor therapy improves renal and hepatic function in patients with cirrhosis secondary to metabolic dysfunction associated steatotic liver disease and type 2 diabetes
Source: Front Endocrinol (Lausanne). 2025 May 15;16:1531295. doi: 10.3389/fendo.2025.1531295 (PMC12119260; doi:10.3389/fendo.2025.1531295)
Supplement: Supplementary file 1 [file DataSheet1.pdf]

	SGLT2i (n)	Insulin (n)	p value
**Diuretics**			
Diuretics $_{\text{baseline}}$	24	22	0.70
Diuretics $_{\text{48 mo}}$	9	25	< 0.01
Diuretics $_{\text{new start}}$	0	5	0.05
Diuretics $_{\text{stopped}}$	15	2	< 0.01
Potassium canrenoate $_{\text{baseline}}$	22	19	0.53
Furosemide $_{\text{baseline}}$	22	22	1.00
Furosemide + Potassium canrenoate $_{\text{baseline}}$	20	19	1.00
Potassium canrenoate $_{\text{48 mo}}$	9	22	< 0.01
Furosemide $_{\text{48mo}}$	9	25	< 0.01
Furosemide + Potassium canrenoate $_{\text{48mo}}$	9	22	< 0.01
Potassium canrenoate $_{\text{new start}}$	0	5	0.05
Furosemide $_{\text{new start}}$	0	5	0.05
Furosemide + Potassium canrenoate $_{\text{new start}}$	0	5	0.05
Potassium canrenoate $_{\text{stopped}}$	13	2	< 0.01
Furosemide $_{\text{stopped}}$	13	2	< 0.01
Furosemide + Potassium canrenoate $_{\text{stopped}}$	11	2	< 0.01
**Beta-blocker**			
Beta-blocker $_{\text{baseline}}$	21/27	21/27	1.00
Beta-blocker $_{\text{48 mo}}$	10/27	21/27	< 0.01
Beta-blocker $_{\text{new start}}$	0/27	1/27	1.00
Beta-blocker $_{\text{stopped}}$	11/27	1/27	< 0.01
**ACEi**			
ACEi $_{\text{baseline}}$	15/27	21/27	0.15
ACEi $_{\text{48 mo}}$	14/27	21/27	0.09
ACEi $_{\text{new start}}$	0/27	0/27	N/A
ACEi $_{\text{stopped}}$	1/27	0/27	1.00
**ARB**			
ARB $_{\text{baseline}}$	3/27	2/27	1
ARB $_{\text{48 mo}}$	3/27	2/27	1
ARB $_{\text{new start}}$	0/27	0/27	N/A
ARB $_{\text{stopped}}$	0/27	0/27	N/A

Supplemental table 1. Longitudinal summary of medication use in both treatment groups. Diuretic use is categorized by specific drug type (potassium canrenoate and/or furosemide) and reported at baseline, study end (48 months), new initiations during the study, and discontinuations. Beta-blockers, ACE inhibitors (ACEi), and angiotensin receptor blockers (ARBs) are similarly reported. Data are presented as patient counts out of total group size (n = 27) along with corresponding p-values for group comparisons.
