# Supplementary material for: Sodium-glucose cotransporter-2 inhibitor therapy improves renal and hepatic function in patients with cirrhosis secondary to metabolic dysfunction associated steatotic liver disease and type 2 diabetes
Source: Front Endocrinol (Lausanne). 2025 May 15;16:1531295. doi: 10.3389/fendo.2025.1531295 (PMC12119260; doi:10.3389/fendo.2025.1531295)
Supplement: Supplementary file 2 [file DataSheet2.pdf]

Comorbidity	SGLT2i (n)	Insulin (n)	p value
Chronic Kidney Disease (CKD)	27	27	1.00
Hypertension	15	15	1.00
Hyperlipidemia	5	7	0.74
Coronary Artery Disease (CAD)	4	6	0.72
Diabetic Neuropathy	3	4	1.00
Diabetic Retinopathy	3	2	1.00

Supplemental table 2. Baseline prevalence of comorbidities in patients treated with SGLT2 inhibitors or insulin. Values are expressed as patient counts out of the total cohort (n = 27 per group). p values reflect group comparisons for each comorbidity.
