# Supplementary material for: Sodium-glucose cotransporter-2 inhibitor therapy improves renal and hepatic function in patients with cirrhosis secondary to metabolic dysfunction associated steatotic liver disease and type 2 diabetes
Source: Front Endocrinol (Lausanne). 2025 May 15;16:1531295. doi: 10.3389/fendo.2025.1531295 (PMC12119260; doi:10.3389/fendo.2025.1531295)
Supplement: Supplementary file 3 [file DataSheet3.pdf]

3a.

Model Component	Description	Mean ± S.E.	F Statistics	p value
Fixed Effects				
Intercept	baseline GFR value	53.3 ± 0.8	900.2	<0.01
Medication	Adjusted mean difference in GFR over 48 months	7.3 ± 2.2	10.5	<0.01
Time	Change in GFR over time	-	6.0	<0.01
$MELDNa_{baseline}$	Effect of baseline MELD-Na of GFR	-	5.2	<0.01
$Albumin_{baseline}$	Effect of baseline albumin on GFR	-	2.0	0.07
$Ascites_{baseline}$	Effect of baseline ascites on GFR	-	0.2	0.62
Medication*time	Interaction between time and medication	-	31.5	<0.01

3b.

Model Component	Description	Mean ± S.E.	F Statistics	p value
Fixed Effects				
Intercept	Baseline GFR value	-	1048.0	< 0.001
Medication	Adjusted mean difference in GFR over 48 months	4.7 ± 0.8	35.4	< 0.001
Time	Change in GFR over time	-	18.1	< 0.001
$MELD3.0_{baseline}$	Effect of baseline MELD 3.0 on GFR	-	1.6	0.14
$Ascites_{baseline}$	Effect of baseline ascites on GFR	-	0.04	0.85
Medication*time	Interaction between time and medication	-	30.0	< 0.001

Supplemental tables 3a–3b. Mixed linear model analysis comparing the effects of SGLT2i and insulin on glomerular filtration rate (GFR) over 48 months. Tables 3 a presents results using baseline MELD-Na, while Tables 3b uses baseline MELD 3.0. Tables 3a and 3b display fixed effects of the covariates medication, time, their interaction, baseline ascites, and MELD score (Na or 3.0) on the outcome variable GFR.
