# Supplementary material for: Sodium-glucose cotransporter-2 inhibitor therapy improves renal and hepatic function in patients with cirrhosis secondary to metabolic dysfunction associated steatotic liver disease and type 2 diabetes
Source: Front Endocrinol (Lausanne). 2025 May 15;16:1531295. doi: 10.3389/fendo.2025.1531295 (PMC12119260; doi:10.3389/fendo.2025.1531295)
Supplement: Supplementary file 4 [file DataSheet4.pdf]

Transition	SGLT2i, n	Insulin, n	p-value
CKD 2 → CKD 2	12	11	1.00
CKD 3a → CKD 2	9	2	0.04
CKD 3b → CKD 2	2	0	0.49
CKD 3a → CKD 3a	3	9	0.10
CKD 3b → CKD 3a	1	0	1.00
CKD 3a → CKD 3b	0	4	0.11
CKD 3b → CKD 3b	0	1	1.00

Supplemental table 4. Transitions of CKD stage over the 48-month study period. This table summarizes patients' transitions between CKD stages from baseline to month 48 for both the SGLT2i and insulin groups. Patient counts for each transition and p-values for between-group comparisons of each transition type are provided.
