# Supplementary material for: Sodium-glucose cotransporter-2 inhibitor therapy improves renal and hepatic function in patients with cirrhosis secondary to metabolic dysfunction associated steatotic liver disease and type 2 diabetes
Source: Front Endocrinol (Lausanne). 2025 May 15;16:1531295. doi: 10.3389/fendo.2025.1531295 (PMC12119260; doi:10.3389/fendo.2025.1531295)
Supplement: Supplementary file 5 [file DataSheet5.pdf]

5a.

Model Component	Description	Mean ± S.E.	F Statistics	p value
Fixed Effects				
Intercept	Baseline LS value	31.4 ± 2.6	398.7	< 0.01
Medication	Adjusted mean difference in LS over 48 months	- 11.1 ± 3.7	15.7	< 0.01
Time	Change in LS over time	-	2.0	0.04
$BMI_{baseline}$	Effect of baseline BMI on LS	-	2.4	0.53
$MELDNa_{baseline}$	Effect of baseline MELDNa of LS	-	1.7	0.18
Medication*time	Interaction between time and medication	-	39.0	< 0.01

5b.

Model Component	Description	Mean ± S.E.	F Statistics	p value
Fixed Effects				
Intercept	Baseline LS value	31.6 ± 1.7	426.7	< 0.01
Medication	Adjusted mean difference in LS over 48 months	- 11.0 ± 2.4	20.3	0.05
Time	Change in LS over time	-	2.0	0.04
$MELDNa_{baseline}$	Effect of baseline MELDNa of LS	-	44.3	<0.01
$BMI_{baseline}$	Effect of baseline BMI on LS	-	14.3	0.06
BMI_{change}	Effect of change in BMI on LS	-	11.3	0.08
Medication*time	Interaction between time and medication	-	39.0	< 0.01

5c.

Model Component	Description	Mean ± S.E	F Statistics	p value
Fixed Effects				
Intercept	Baseline LS value	-	449.2	< 0.01
Medication	Adjusted mean difference in LS over 48 months	−11.2 ± 2.7	17.2	< 0.01
Time	Change in LS over time	-	2.0	0.04
$BMI_{baseline}$	Effect of baseline BMI on LS	-	1.1	0.44
$MELD3.0_{baseline}$	Effect of baseline MELD 3.0 on LS	-	1.1	0.41
Medication*time	Interaction between time and medication	-	38.8	< 0.01

5d

Model Component	Description	Mean ± S.E.	F Statistics	p value
Fixed Effects				
Intercept	Baseline LS value	-	568.5	0.02
Medication	Adjusted mean difference in LS over 48 months	−11.0 ± 2.9	14.6	0.16
Time	Change in LS over time	-	2.1	0.04
$MELD3.0_{baseline}$	Effect of baseline MELD 3.0 on LS	-	21.5	< 0.01
$BMI_{baseline}$	Effect of baseline BMI on LS	-	9.8	< 0.01
BMI_{change}	Effect of change in BMI on LS	-	8.8	< 0.01
Medication*time	Interaction between time and medication	-	39.0	< 0.01

Supplemental Tables 5a–5d. Mixed linear model analysis comparing the effects of SGLT2i and insulin on liver stiffness (LS) over 48 months. Tables 5a and 5b present results using baseline MELD-Na, while Tables 5c and 5d use baseline MELD 3.0. Tables 5a and 5c display fixed effects of the covariates medication, time, their interaction, baseline BMI, and MELD score (Na or 3.0) on LS. Tables 5b and 5d further adjust for the change in BMI between the start and end of the study. In both MELD-Na and MELD 3.0 models, the addition of BMI change attenuated the effect of medication type on LS, though the interaction between medication and time remained statistically significant across all models.
