# Supplementary material for: Sodium-glucose cotransporter-2 inhibitor therapy improves renal and hepatic function in patients with cirrhosis secondary to metabolic dysfunction associated steatotic liver disease and type 2 diabetes
Source: Front Endocrinol (Lausanne). 2025 May 15;16:1531295. doi: 10.3389/fendo.2025.1531295 (PMC12119260; doi:10.3389/fendo.2025.1531295)
Supplement: Supplementary file 6 [file DataSheet6.pdf]

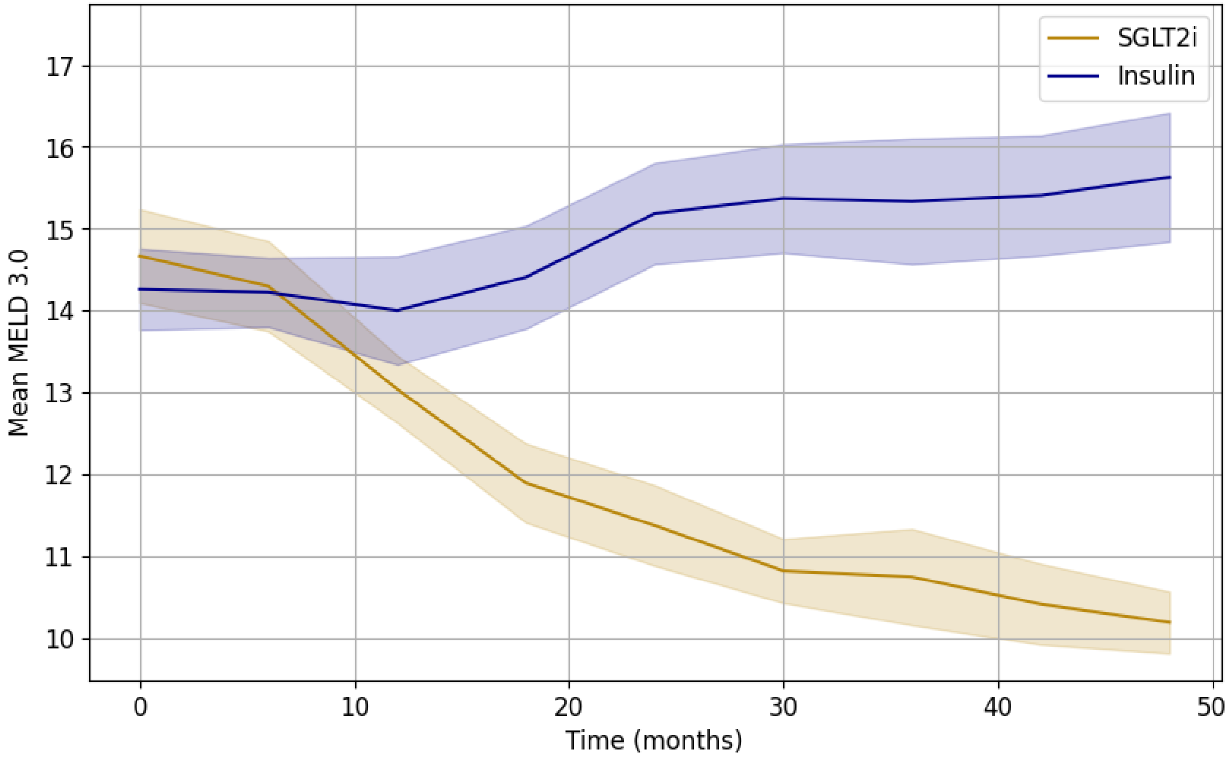


| Time | 0 mo | 6 mo | 12 mo | 18 mo | 24 mo | 30 mo | 36 mo | 42 mo | 48 mo |
|---|---|---|---|---|---|---|---|---|---|
| SGLT2i mean, 95% CI | 14.7<br>14.1-15.2 | 14.3<br>13.7-14.8 | 13.0<br>12.6-13.4 | 11.9<br>11.4-12.4 | 11.4<br>10.9-11.9 | 10.8<br>10.4-11.2 | 10.7<br>10.2-11.3 | 10.4<br>9.9-10.9 | 10.2<br>9.8-10.6 |
| Insulin mean, 95% CI | 14.3<br>13.8-14.8 | 14.2<br>13.8-14.6 | 14.0<br>13.3-14.7 | 14.4<br>13.8-15.0 | 15.2<br>14.6-15.8 | 15.4<br>14.7-16.0 | 15.3<br>14.6-16.1 | 15.4<br>14.7-16.1 | 15.6<br>14.8-16.4 |
| p value | 0.18 | 0.75 | 0.01 | <0.01 | <0.01 | <0.01 | <0.01 | <0.01 | <0.01 |

**Supplemental figure 1.** Representation of MELD 3.0 changes over time for SGLT2i and insulin groups. Results of independent T test analysis comparing the mean MELD 3.0 at 6 month intervals for the two groups are provided.
