# Supplementary material for: Sodium-glucose cotransporter-2 inhibitor therapy improves renal and hepatic function in patients with cirrhosis secondary to metabolic dysfunction associated steatotic liver disease and type 2 diabetes
Source: Front Endocrinol (Lausanne). 2025 May 15;16:1531295. doi: 10.3389/fendo.2025.1531295 (PMC12119260; doi:10.3389/fendo.2025.1531295)
Supplement: Supplementary file 7 [file DataSheet7.pdf]

6a.

Variable	SGLT2i (mean ± se)	p SGLT2i (0 vs 48)	Insulin (mean ± se)	p Insulin (0 vs 48)	p (SGLT2i vs Insulin)	p (Δ SGLT2i vs Insulin)
Na $_{0\ mo}$	139.8 ± 0.5	0.63	139.4 ± 0.2	0.09	0.43	
Na $_{48\ mo}$	139.6 ± 0.3		138.7 ± 0.4		0.09	
Na $_{\Delta\ (48\ -\ 0)}$	-0.2 ± 0.5		-0.7 ± 0.4			0.48
Tbili $_{0\ mo}$	2.4 ± 0.1	< 0.01	2.5 ± 0.1	<0.01	0.18	
Tbili $_{48\ mo}$	1.9 ± 0.0		2.7 ± 0.1		< 0.01	
Tbili $_{\Delta\ (48\ -\ 0)}$	-0.5 ± 0.1		0.2 ± 0.1			< 0.01
Cr $_{0\ mo}$	1.2 ± 0.0	<0.01	1.1 ± 0.0	<0.01	0.31	
Cr $_{48\ mo}$	1.0 ± 0.0		1.2 ± 0.0		< 0.01	
Cr $_{\Delta\ (48\ -\ 0)}$	-0.2 ± 0.0		0.1 ± 0.0			< 0.01
INR $_{0\ mo}$	1.2 ± 0.0	<0.01	1.2 ± 0.0	0.1	0.62	
INR $_{48\ mo}$	1.0 ± 0.0		1.2 ± 0.0		< 0.01	
INR $_{\Delta\ (48\ -\ 0)}$	-0.2 ± 0.0		0.0 ± 0.0			< 0.01
Albumin $_{0\ mo}$	3.0 ± 0.0	<0.01	2.9 ± 0.0	0.04	0.37	
Albumin $_{48\ mo}$	3.3 ± 0.0		3.0 ± 0.0		< 0.01	
Albumin $_{\Delta\ (48\ -\ 0)}$	0.3 ± 0.0		-0.1 ± 0.0			< 0.01

6b.

Variable	Time	SGLT2i Median (Range)	Insulin Median (Range)	p value
Ascites	0 mo	2 (1, 2)	2 (1, 2)	0.4
Ascites	48 mo	1 (1, 1)	1 (1, 2)	< 0.01
HE	0 mo	2 (1, 2)	1 (1, 2)	0.03
HE	48 mo	1 (1, 1)	1 (1, 2)	< 0.01
Bilirubin	0 mo	2 (1, 2)	2 (2, 2)	0.34
Bilirubin	48 mo	1 (1, 2)	2 (2, 3)	< 0.01
Albumin	0 mo	2 (2, 3)	2 (2, 2)	0.08
Albumin	48 mo	2 (1, 2)	2 (2, 3)	0.05
INR	0 mo	1 (1, 1)	1 (1, 1)	1.0
INR	48 mo	1 (1, 1)	1 (1, 1)	1.0

Supplemental table 6a. Mean ± standard error (SE) of each MELD and MELD 3.0 variable (serum sodium, creatinine, bilirubin, INR, and albumin) are provided at baseline (0 months) and at 48 months for both treatment groups. p-values are provided for: (1) within-group changes over time, (2) between-group comparisons at each time point, and (3) between-group comparison of changes over time.

Supplemental table 6b. Summarizes median (range) of Child-Pugh subcomponent points at baseline and 48 months in each group, with associated p-values for between-group comparisons at each time point.
