# Supplementary material for: Sodium-glucose cotransporter-2 inhibitor therapy improves renal and hepatic function in patients with cirrhosis secondary to metabolic dysfunction associated steatotic liver disease and type 2 diabetes
Source: Front Endocrinol (Lausanne). 2025 May 15;16:1531295. doi: 10.3389/fendo.2025.1531295 (PMC12119260; doi:10.3389/fendo.2025.1531295)
Supplement: Supplementary file 8 [file DataSheet8.pdf]

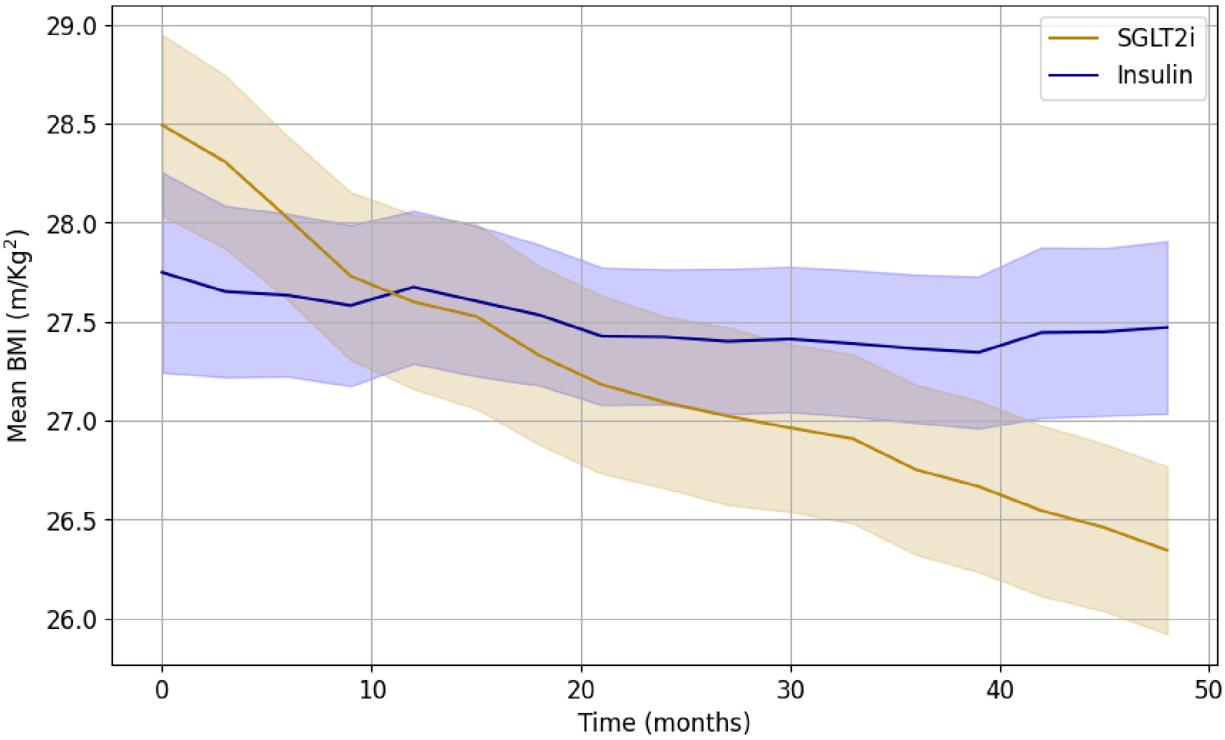


| time | 0 mo | 6 mo | 12 mo | 18 mo | 24 mo | 30 mo | 36 mo | 42 mo | 48 mo |
|---|---|---|---|---|---|---|---|---|---|
| SGLT2i mean, 95% CI | 28.4 28.1-28.7 | 27.9 27.6-28.2 | 27.6 26.2-27.9 | 27.2 26.9-27.6 | 27.1 26.7-27.4 | 26.9 26.6-27.2 | 26.7 26.4-27.0 | 26.5 26.2-26.8 | 26.3 25.9-26.8 |
| Insulin mean, 95% CI | 27.7 27.4-28.0 | 27.6 27.3-27.9 | 27.6 27.4-27.9 | 27.5 27.2-27.7 | 27.4 27.2-27.7 | 27.4 27.1-27.7 | 27.3 27.1-27.6 | 27.4 27.1-27.7 | 27.5 27.1-27.9 |
| p value | 0.04 | 0.20 | 0.70 | 0.30 | 0.08 | 0.02 | <0.01 | <0.01 | <0.01 |

**Supplemental figure 2.** Representation of BMI changes over time for SGLT2i and insulin groups. Results of independent T test analysis comparing the mean BMI at 6 month intervals for the two groups are provided.
