# Supplementary material for: Sodium-glucose cotransporter-2 inhibitor therapy improves renal and hepatic function in patients with cirrhosis secondary to metabolic dysfunction associated steatotic liver disease and type 2 diabetes
Source: Front Endocrinol (Lausanne). 2025 May 15;16:1531295. doi: 10.3389/fendo.2025.1531295 (PMC12119260; doi:10.3389/fendo.2025.1531295)
Supplement: Supplementary file 9 [file DataSheet9.pdf]

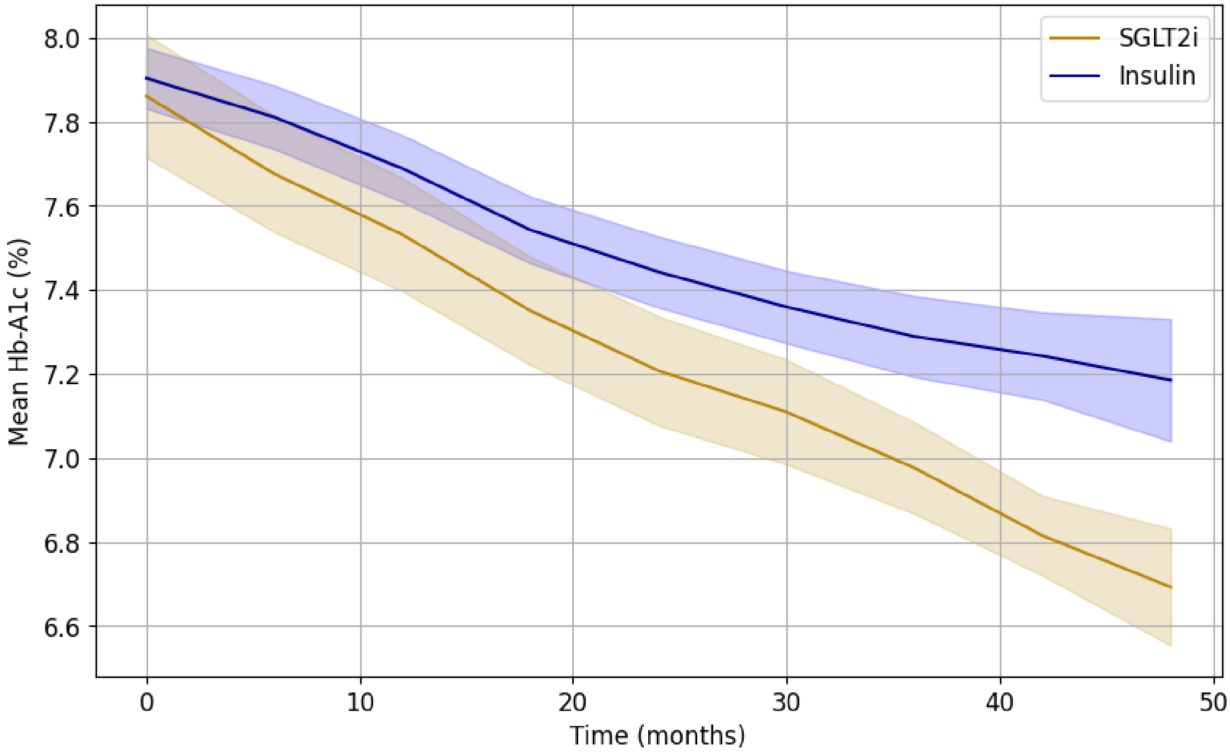

time	0 mo	6 mo	12 mo	18 mo	24 mo	30 mo	36 mo	42 mo	48 mo
SGLT2i mean, 95% CI	7.9 7.7-8.0	7.7 7.7-7.6	7.5 7.4-7.7	7.4 7.2-7.5	7.2 7.1-7.3	7.1 7.0-7.2	7.0 6.9-7.1	6.8 6.9-7.1	6.7 6.6-6.8
Insulin mean, 95% CI	7.9 7.8-8.0	7.8 7.7-7.9	7.7 7.6-7.8	7.5 7.5-7.6	7.4 7.4-7.5	7.4 7.3-7.4	7.3 7.2-7.4	7.2 7.1-7.3	7.2 7.0-7.3
p value	0.8	0.10	0.05	0.01	<0.01	<0.01	<0.01	<0.01	<0.01

Supplemental figure 3. Representation of HbA1c changes over time for SGLT2i and insulin groups. Results of independent T test analysis comparing the mean HbA1c at 6 month intervals for the two groups are provided.
