# Supplementary material for: Sodium-glucose cotransporter-2 inhibitor therapy improves renal and hepatic function in patients with cirrhosis secondary to metabolic dysfunction associated steatotic liver disease and type 2 diabetes
Source: Front Endocrinol (Lausanne). 2025 May 15;16:1531295. doi: 10.3389/fendo.2025.1531295 (PMC12119260; doi:10.3389/fendo.2025.1531295)
Supplement: Supplementary file 10 [file DataSheet10.pdf]

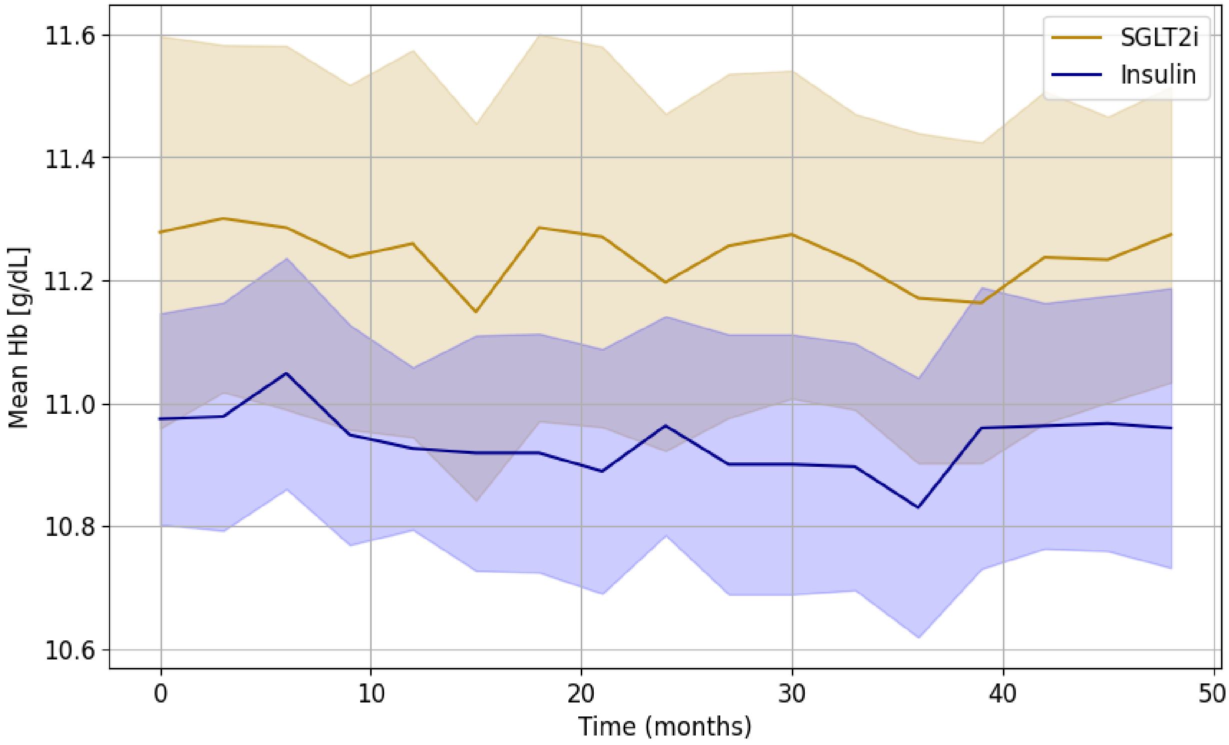

time	0 mo	6 mo	12 mo	18 mo	24 mo	30 mo	36 mo	42 mo	48 mo
SGLT2i mean, 95% CI	11.3 10.9-11.6	11.3 10.9-11.6	11.3 10.9-11.6	11.3 10.9-11.7	11.2 10.9-11.5	11.3 10.9-11.6	11.2 10.8-11.5	11.2 10.9-11.6	11.3 10.9-11.6
Insulin mean, 95% CI	11.0 10.6-11.3	11.0 10.7-11.4	10.9 10.6-11.3	10.9 10.5-11.3	11.0 10.6-11.3	10.9 10.6-11.2	10.8 10.5-11.2	11.0 10.6-11.3	11.0 10.6-11.3
p value	0.11	0.20	0.06	0.06	0.17	0.04	0.06	0.11	0.07

Supplemental figure 4. Representation of Hb changes over time for SGLT2i and insulin groups. Results of independent T test analysis comparing the mean Hb at 6 month intervals for the two groups are provided.
