# Supplementary material for: Sodium-glucose cotransporter-2 inhibitor therapy improves renal and hepatic function in patients with cirrhosis secondary to metabolic dysfunction associated steatotic liver disease and type 2 diabetes
Source: Front Endocrinol (Lausanne). 2025 May 15;16:1531295. doi: 10.3389/fendo.2025.1531295 (PMC12119260; doi:10.3389/fendo.2025.1531295)
Supplement: Supplementary file 11 [file DataSheet11.pdf]

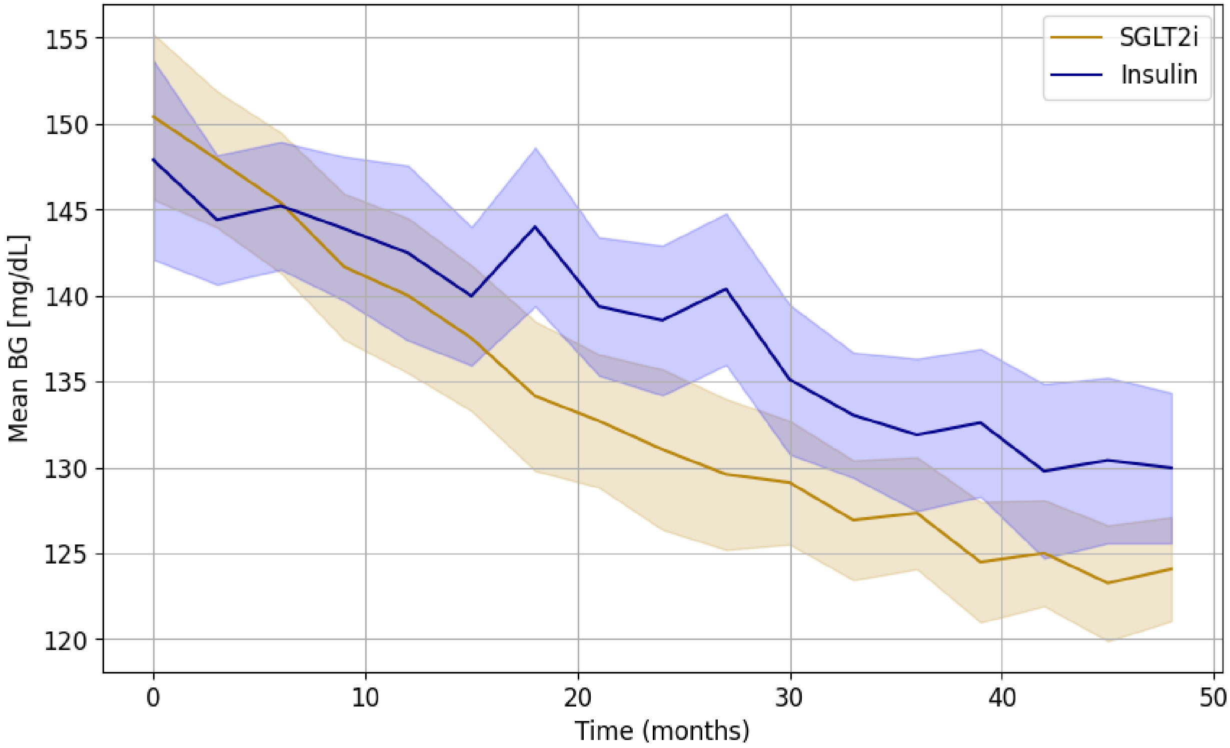

time	0 mo	6 mo	12 mo	18 mo	24 mo	30 mo	36 mo	42 mo	48 mo
SGLT2i mean, 95%CI	150<br>158-143	145<br>140-151	140<br>133-147	134<br>128-140	131<br>125-137	129<br>123-135	127<br>122-133	125<br>119-131	124<br>119-129
Insulin mean, 95%CI	148<br>155-140	145<br>140-151	142<br>138-150	144<br>138-150	139<br>132-145	135<br>129-141	132<br>126-137	130<br>124-136	130<br>125-135
p value	0.52	0.21	0.48	<0.01	0.03	0.04	0.11	0.12	0.04

Supplemental figure 5. Representation of BG changes over time for SGLT2i and insulin groups. Results of independent T test analysis comparing the mean BG at 6 month intervals for the two groups are provided.
