# Supplementary material for: Sodium-glucose cotransporter-2 inhibitor therapy improves renal and hepatic function in patients with cirrhosis secondary to metabolic dysfunction associated steatotic liver disease and type 2 diabetes
Source: Front Endocrinol (Lausanne). 2025 May 15;16:1531295. doi: 10.3389/fendo.2025.1531295 (PMC12119260; doi:10.3389/fendo.2025.1531295)
Supplement: Supplementary file 12 [file DataSheet12.pdf]

	Time 0 (mean ± se)	Time 48 (mean ± se)	p value
total insulin, U/kg/d	0.49 ± 0.02	0.51 ± 0.01	0.06
total insulin, U/d	38.0 ± 1.4	38.8 ± 1.0	0.49
basal insulin, U/d	19.1 ± 1.5	20.5 ± 1.3	0.14
rapid insulin, U/d	18.9 ± 0.6	18.3 ± 0.7	0.48

Supplemental table 7. Summary of insulin dosing at baseline (Time 0) and at the end of the study period (48 months) in the insulin-treated cohort. Values are presented as mean ± standard error (SE) for total daily insulin use in units per kilogram of body weight (U/kg), total daily insulin use (U/day), basal insulin (U/day), and rapid-acting insulin (U/day). No statistically significant differences in insulin dosing were observed over the study period. Glargine Toujeo was used as the basal insulin, and Lispro Humalog was the rapid-acting insulin administered.
